# In situ detection of dead cells from live cells via a DC plus low frequency AC resistive pulse sensor

**DOI:** 10.1007/s10544-026-00797-y

**Published:** 2026-02-13

**Authors:** Parker Lybrook, Heyi Chen, Emma Barna, Jacob Brown, Ashley Wong, Joseph Ketchum, Ge Zhang, Jiang Zhe

**Affiliations:** 1 Department of Mechanical Engineering, University of Akron, Akron, OH 44325 USA; 2 Department of Biomedical Engineering, University of Akron, Akron, OH 44325 USA

**Keywords:** Cell analysis, Microchannel, Multispectral, Cell viability

## Abstract

Differentiation and detection of live and dead cells are critical for assessing cell viability in biomedical research, evaluating drug efficacy, and monitoring cytotoxicity in therapeutic applications. We present a microfluidic sensor that consists of two successive resistive pulse sensing channels. An excitation signal composed of a low-frequency AC (75 kHz) component and a DC bias was used to measure four key parameters. Through the AC measurement, differences in cell impedance causes variations in phase angle and voltage peak. From the DC measurement, cell size can be inferred from the resistive pulse magnitude, and the cell’s zeta potential is represented by the transit time difference. Human umbilical vein endothelial cells (HUVECs) and human mesenchymal stem cells (hMSCs) were used to demonstrate the device’s utility. A soft margin support vector machine (SVM) was applied to define the decision boundary based on analysis of the four parameters. For both cell types, live and dead cells formed distinct clusters, achieving maximum classification accuracies of up to 100%. Additionally, HUVECs treated with either ethanol or staurosporine (STS) were classified with accuracies up to 100%. Compared to previous microfluidic resistive pulse sensor (RPS), this approach can determine cell viability without the need for complex labeling or modifications. Unlike impedance cytometry, it does not require high-frequency measurements, significantly reducing hardware requirements and data processing complexity, while still providing multiparametric measurements of cells. These measurements allow the use of soft SVM to classify cell groups with higher accuracy than single-parameter differentiation.

**Supplementary Information:**

The online version contains supplementary material available at 10.1007/s10544-026-00797-y.

## Introduction

Cell viability testing is a fundamental assessment in biomedical research because it directly reflects the health, survival, and functional status of cells under experimental or therapeutic conditions (Madorran et al. 2025). By quantifying the proportion of live cells, viability assays provide critical insights into how cells respond to environmental factors, drug treatments, or biomaterial interactions (Gordon et al. 2018; Płuciennik et al. 2024; Samuel et al. 2025). These measurements are essential for optimizing culture conditions, ensuring experimental reproducibility, and validating the safety and effectiveness of emerging drugs and biomaterials (Kamiloglu et al. 2020; Adan et al. 2016). Accurate discrimination between live and dead cells is particularly important because dead cells can release intracellular contents, alter the microenvironment, and interfere with downstream analyses, leading to misleading interpretations if not properly identified and excluded (Madorran et al. 2025; Adan et al. 2016).

A wide range of cell viability assays have been developed to distinguish live cells from dead or dying populations using principles such as membrane integrity, metabolic activity, enzymatic release, or fluorescent labeling (Kamiloglu et al. 2020; Aslantürk 2018; Madorran et al. 2025). Membrane-integrity assays, including trypan blue exclusion and Live/Dead staining, are simple and widely accessible but are typically destructive and limited to endpoint analysis (Hu et al. 2022; Aslantürk 2018; Madorran et al. 2025). Metabolic assays such as MTT, XTT, Alamar Blue, and ATP quantification are convenient and scalable; however, they provide only indirect measures of viability, as metabolic activity can fluctuate due to stress, cell cycle stage, or environmental conditions, potentially leading to inaccurate estimates of live cell numbers (Ghasemi et al. 2021; Aslantürk 2018; Meerloo et al. 2011; Kumar & Nagarajan 2018). Enzyme-based cytotoxicity assays, such as LDH release, offer non-destructive detection of membrane damage but cannot directly quantify live cells and may accumulate background signal over time (Aslantürk 2018; Madorran et al. 2025). Imaging-based and flow cytometry approaches provide high sensitivity and single-cell resolution, yet they often require labeling, cell dissociation, or repeated dye exposure, which can disrupt physiological conditions and limit longitudinal monitoring (Drescher et al. 2021; Murali et al. 2019). Although these approaches are valuable and well-established, they are frequently destructive, endpoint-limited, dye-dependent, and poorly suited for dynamic culture systems. These limitations highlight the need for non-destructive, accurate and sensitive methods for assessing cell viability.

Recently, multi-frequency microfluidic approaches were used to classify and differentiate cells. Cells exhibit frequency-dependent electrical characteristics (e.g. impedance, phase angle, etc.) under alternating current (AC) excitations. Sui et al. (2020) and Gawad et al. (2001) used a microfluidic device with two excitation frequencies (e.g. 500 kHz as low frequency, 20 MHz as high frequency) to differentiate live cells from dead cells through peak changes of output voltages at the two frequencies. Eades et al. (2023) also used multiple AC frequencies (e.g.1.0 MHz, 1.5 MHz, 1.8 MHz, and 2.0 MHz) to differentiate live and dead cells in terms of changes in impedance magnitude and phase angle. While these multi-frequency microfluidic devices enabled label-free cell detection and characterization (including cell viability), multi-frequency methods (especially at high frequencies over 1 MHz) require complex hardware for data acquisition. Post processing of the large amount of data is challenging for real time cell detection and differentiation. Studies also attempted to extract membrane capacitance and cytoplasmic resistance from multi-frequency measurements (Favakeh et al. 2025). The challenge is that it is difficult to find an equivalent circuit model that can represent the cell’s electrical behavior across different frequency bands.

Microfluidic resistive pulse sensors (RPSs) have been used to measure the size and mobility of various cell types and particles (Ni et al. 2020; Sun et al. 2013). As a cell enters a RPS sensing channel, it distorts the electrical field, causing a resistance change of the sensing channel (i.e. resistive pulse). The magnitude of the pulse represents the cell’s size; the pulse duration reflects the cell’s mobility through the channel, which is affected by the cell’s surface charge or interaction with external fields (Ni et al. 2020). Due to the simple structure of RPS and its ability to provide key parameter measurements on single cells, it has been used for counting and detection of a variety of cells including pathogen, bacteria, pollen, etc. (Varga et al. 2022; Hattori et al. 2020; Jagtiani et al. 2006). Specifically, Han et al. (2016) developed an immunosensor for pathogen detection based on a resistive pulse sensor. Antibody-conjugated microparticles were used to functionalize the capture chamber so that target cells were captured by the chamber. By counting cells at the inlet and outlet of the chamber, target S. cerevisiae cells were detected. Liu et al. (2016) developed a microfluidic RPS in combination with a magnetic bead cell assay. Target cells bound to antibody-functionalized magnetic particles experienced reduced mobility in an external magnetic field and longer transit time; hence by measuring cells’ transit time through a microchannel, target cells can be identified. While these methods can be used for detecting live and dead cells, both require antibody-based surface modifications of the cells or the microchannel.

Here, we report a novel microfluidic sensor capable of differentiating live and dead cells in a mixed population. This device incorporates two consecutive sensing channels. A low-frequency AC (75 kHz) signal combined with a DC bias was used for excitation. The DC bias enables measurement of changes in DC magnitude and transit time, which indicate the cell’s size and zeta potential, while the AC excitation enables measurement of changes in phase angle and AC magnitude, which reflect the cell’s membrane capacitance and impedance. We chose to use a low frequency AC excitation based on a recent finding by Liu et al. (2018), who reported clear differences in phase angle and impedance between cells in normoxic and hypoxic conditions at relatively low frequencies (e.g., ~ 100 kHz level). The dual excitation configuration enables simultaneous measurement of four key properties that are affected by the cell’s intrinsic characteristics: size, surface charge, membrane capacitance and overall impedance. Compared to traditional RPSs, which are designed to measure the size, count and zeta potential of microparticles as well as bio-modified RPSs which can assess cell viability via cell labelling or modifications (e.g. antibody conjugation), our AC-DC approach provides four key measurements. These measurements allow cell viability to be determined using SVM classification with higher accuracy than single-parameter differentiation, without the need for complex labeling or modifications. Impedance cytometry is well known for identifying cell viability or type by measuring impedance characteristics (phase angle, magnitude, etc.) at multiple low and high AC frequencies. In comparison, our approach not only provides multiparametric measurements of cells but eliminates the need for high frequency measurements, significantly reducing the hardware requirements as well as the size and complexity of data processing, thereby facilitating the real time measurement necessary for field applications.

## Device, experiment setup and fabrication

### Device design and principle

**Fig. 1:**
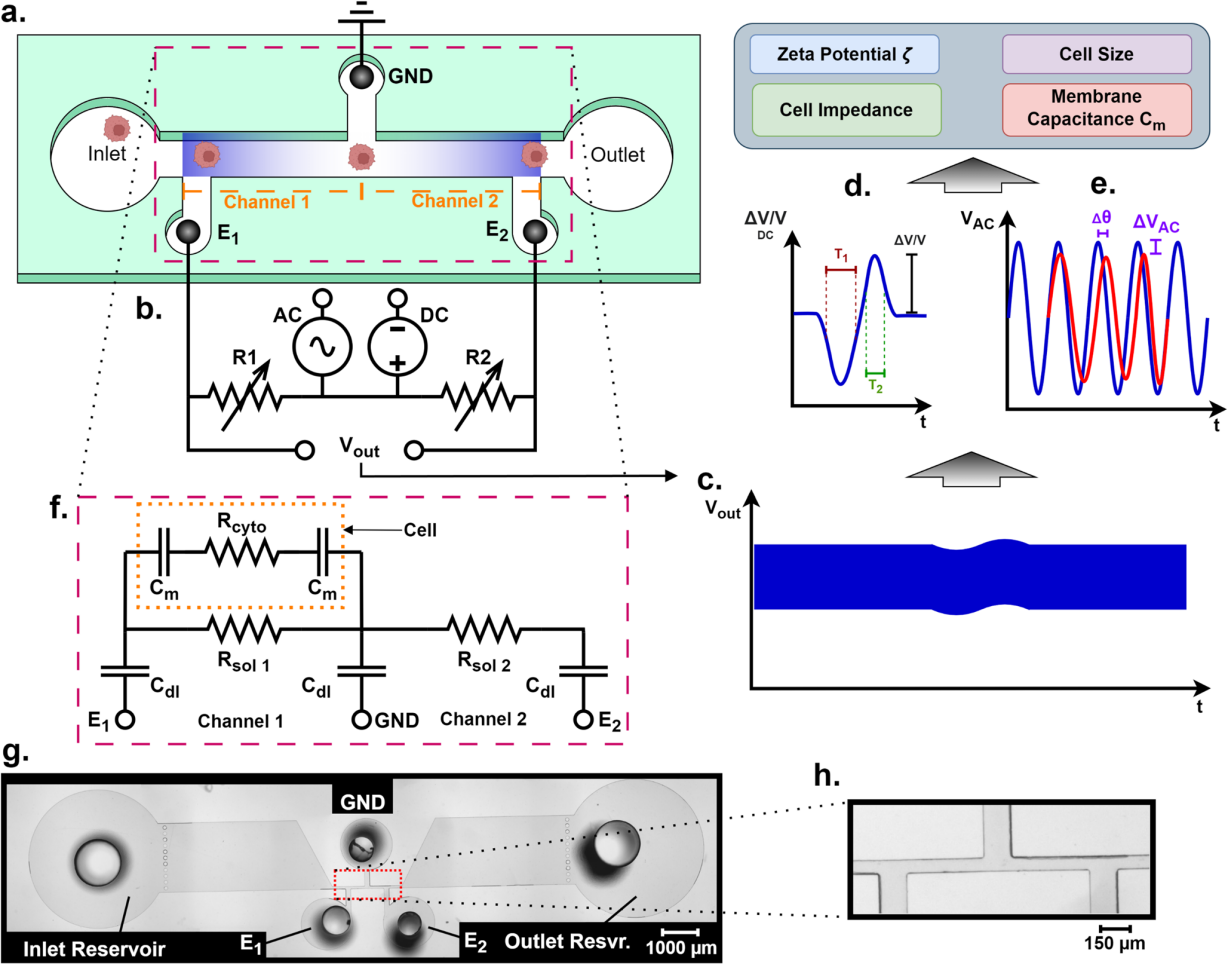
**a**). Schematic of the microfluidic device for in situ, label-free multiparametric measurement of cell properties. **b**). Diagram of the measurement circuit. **c**). Illustration of the voltage output/pulse induced by a cell. **d**). Illustration of the DC voltage pulse component demonstrating the ΔV_DC_ and transit times T_1_ and T_2_. **e**). Illustration of the AC voltage pulse component demonstrating the phase shift θ and ΔV_AC_. **f**). Equivalent circuit model of the detection channel with a cell present in the first sensing channel. **g**). Composite microscopic image of the device. **h**). Magnified image of the sensing channels

Figure 1a shows the schematic of the device, which consists of two consecutive sensing microchannels and three Ag/AgCl electrodes secured in the three electrode holes. An AC plus DC combined excitation is applied to electrodes E1 and E2 while the GND electrode is grounded. Figure 1b shows the diagram of the measurement circuit. Under the excitation signal, a cell passing through the device causes an impedance change of both channels 1 and 2 and generates two consecutive voltage pulses (see Figs. 1c).

When a DC voltage is applied, a negatively charged cell in channel 1 is decelerated through electrophoresis; therefore, the transit time T_1_ would be longer than without a DC bias. When the cell passes through channel 2, it is accelerated and causes a shorter transit time, T_2_ (see Fig. 1d). The difference between transit times T_1_ and T_2_ reflects the surface charge of the cell. In this article, (1/T_2_−1/T_1_) is utilized because it is proportional to the cell’s zeta potential (Ni et al. 2020):

1 $$\:\zeta\:=\frac{\eta\:l}{2E{\epsilon\:}_{r}{\epsilon\:}_{0}}\left(\frac{1}{{T}_{2}}-\frac{1}{{T}_{1}}\right)$$

where η is the viscosity of the aqueous solution, E is the electric field (E = V_e_/l), ε is the permittivity, and l is the length of the sensing channel.

In addition, the magnitude of the voltage/resistance pulse induced by a cell (see Fig. 1d) reflects the cell size (DeBlois & Bean, 1970) :

2 $$\:\frac{\varDelta\:R}{R}=\frac{{d}^{3}}{{D}^{2}L}(\frac{{D}^{2}}{2{L}^{2}}+\frac{1}{\sqrt{1+{\left(\frac{D}{L}\right)}^{2}}})F\left(\frac{{d}^{3}}{{D}^{3}}\right)$$

where $\:F\left(\frac{{d}^{3}}{{D}^{3}}\right)$ is a correction factor, d is the diameter of the cell, L is the length of the channel, and D is the diameter of the channel. Hence the DC measurement would indicate the cell surface charge and size. Compared to live cells, dead cells typically exhibit changes in the surface charge or size (Lapizco-Encinas 2024; Hughes 2024; Ayala-Torres et al. 2014; Klassen et al. 1993; Kato et al. 2021; Bondar et al. 2012) meaning they can be used as two characteristics for differentiation.

Under the AC excitation, a cell can be modeled electrically by two membrane capacitances (C_m_), and a cytoplasm resistance (R_cyto_). The presence of a cell in the sensing channel would cause a change in the phase angle Δθ (affected by C_m_), and ΔV_AC_/V_AC_ (affected by cell impedance), as shown in Fig. 1e. An equivalent circuit model obtained from Foster and Schwan’s simplified model (commonly used for single cell impedance analysis) is shown in Fig. 1f. where R_sol_ and C_dl_ represent the electrolyte resistance and the electrodes’ double layer capacitance (Morgan et al. 2007; Gawad et al. 2001, 2004; Sun et al. 2007). At certain frequencies, membrane capacitance (C_m_) and cytoplasm resistance (R_cyto_), play a critical role in the overall impedance. C_m_ depends on the membrane’s integrity and composition as well as the membrane’s relative permittivity (Favakeh et al. 2025; Eades et al. 2023). Live and dead cells exhibit substantial differences in their membrane properties. Dead cells often have compromised membranes integrity or lipid disorganization, which reduce effective insulating behaviors and diminish ion polarization effects (Eades et al. 2023). These membrane disruptions typically lead to reduced C_m_ compared to live cells (Favakeh et al. 2025; Eades et al. 2023; Lapizco-Encinas 2024). Additionally, R_cyto_ is affected by intracellular ionic content and ion exchange with the surrounding medium (Favakeh et al. 2025). Dead cells have increased ion exchanges with the surrounding medium due to increases in membrane permittivity, causing greater changes in intracellular ionic content and the loss of ionic homeostasis (Favakeh et al. 2025). Therefore, dead cells would exhibit different intracellular conductivity and R_cyto_ compared to live cells. The difference in C_m_ and R_cyto_ between live and dead cells would generate different Δθ and ΔV_AC_/V_AC_ values in AC measurements. As a result, live and dead cells can be distinguished by measuring the Δθ, and ΔV_AC_/V_AC_ as cells pass through the sensing channels. A recent study revealed adequate Δθ, and ΔV_AC_/V_AC_ measurements at a low frequency of ~ 100 kHz (Liu et al. 2018). Thus, a combination of low frequency AC and DC measurements with this device enables quantitative characterization of the transit time difference (1/T_2_−1/T_1_) (reflecting the cell’s surface charge), DC pulse magnitude (reflecting cell size), phase angle, and AC voltage magnitude (affected by C_m_ and the cell’s overall impedance). Using this comprehensive multiparametric evaluation, live and dead cells can be readily differentiated. This approach does not require surface modification or labeling, and the cells remain intact for downstream analyses or applications after measurement. Figures 1g and h show a composite microscopic image of the device and a magnified view of the sensing channels, respectively.

### Device fabrication

The microfluidic device was manufactured using the standard soft lithography method. First, the mold for the sensing channels and the reservoirs was patterned by spin coating a 40-µm thick SU-8 2025 negative photoresist (MicroChem, MA, USA) on a 4-inch silicon wafer in three steps (500 rpm for 10 s, 2000 rpm for 35 s, and 1000 rpm for 15 s). After a soft bake at 95 °C for 6 min, photolithography was applied with a UV exposure of 160 mJ/cm^2^, which was followed by a 5-minute post-exposure bake at 95 °C. SU-8 developer was used to develop the mold through a 3-minute immersion then an ethanol and DI water rinse was applied. To complete the fabrication of the micro mold, the wafer received a final hard bake at 180 °C for 2 hours.

Next, polydimethylsiloxane (PDMS, Dow Corning Sylgard 184 Silicone Elastomer Kit) was mixed and poured onto the mold. The PDMS was degassed under a −25 inHg vacuum and then cured at 65 °C for 3 h in a vacuum oven (Fisher Scientific ™, Isotemp Vacuum Oven Model 280 A). After cooling, the mold was demolded from the silicon wafer, and the inlet/outlet reservoirs and electrode holes were punched using a 1.5 mm and a 1.0 mm biopsy punch respectively. Lastly, the molded PDMS was treated using air plasma at 200 mTorr and 100 W for 35 s and bonded to a microscope glass slide (Fisher Scientific ™, Premium Plain Glass Microscope Slides). Note that prior to bonding, the dimensions of the sensing channel were measured using a surface profilometer (Dektak 150, Veeco Instrument, NY, USA). The channels had a measured dimension of 39.657 ± 0.223 μm in height, 67.311 ± 0.754 μm in width, and 535.540 ± 2.526 μm in length per channel, in contrast to a nominal dimension of 40 μm in height, 75 μm in width, and 550 μm in length per channel.

### Cell culture and preparation

Human umbilical vein endothelial cells (HUVECs) and human mesenchymal stem cells (hMSCs) were purchased from Lonza (Walkersville, MD). HUVECs were cultured using the EGM-2 BulletKit (Lonza, Walkersville, MD) supplemented with 1% penicillin–streptomycin (P/S). hMSCs were cultured in Gibco Dulbecco’s Modified Eagle Medium (DMEM) (low glucose) (Gibco Inc., Watham, MD) supplemented with 10% fetal bovine serum (FBS) and 1% P/S. All cells were maintained in a humidified cell culture incubator at 37 °C with 5% CO_2_. Culture media was changed every other day for HUVECs and every 3 days for hMSCs. All experiments were performed with passage 11 HUVECs and hMSCs.

Cellular treatments were optimized to achieve 0% viability across all dead cell samples, as confirmed by either Trypan Blue exclusion assay (ethanol treatment group) or cellular morphology analysis (STS treatment group). Necrotic cell death was induced using 70% ethanol. Briefly, culture medium was removed, cells were washed with PBS and then treated with 70% ethanol for 5 min at room temperature. Complete cell death was confirmed using a Trypan Blue exclusion assay. Apoptotic cell death was induced using 200 nM staurosporine (STS; Millipore-Sigma, Burlington, MA). Cells were incubated in culture medium containing 200 nM STS for 1 h, and complete apoptotic cell death was confirmed by microscopic analysis of late-stage apoptotic cellular morphology. Following viability assessments, cells were resuspended in culture media at a concentration of 12,500 cells/ml. Mixed samples (used in Section 3.3) were prepared by combining live and dead cells at predetermined ratios.

### Measurement setup

Prior to each test, the device was thoroughly cleaned using ethanol and DI water. The Ag/AgCl electrodes were inserted into the electrode holes and secured via an interference fit between the 1.0 mm electrode holes and the 1.5 mm electrodes. Next, residual DI water and air were purged from the device with cell culture media.

Each cell suspension sample was injected into a prefilled feeding pipe connected to the inlet reservoir of the device. A flow controller (Flow EZ, Fluigent) was used to apply a constant pressure of 1 kPa across the device to generate a constant flow rate for every test.

The excitation signal, composed of a DC signal of 4 V and an AC signal (peak to peak voltage of 400 mV at 75 kHz), was applied to electrodes E1 and E2. A Wheatstone bridge setup was used to measure the voltage difference between the two channels (shown in Fig. 1), R1 and R2 values were set as ~ 100 kΩ to balance the resistance of the channels. The Wheatstone bridge output was amplified by a differential amplifier (AD620BN, Analog Devices) with gain resistor set to 1 kΩ for a gain of 50.4. This amplified voltage output was then recorded using a DAQ board (NI USB-6361, National Instruments) and accompanying software (LabVIEW, National Instruments) at a 1.2 MHz sampling rate. The collected data was post processed with a custom MATLAB script.

## Results and discussion

### Differentiating Live/Dead HUVECs

Two batches of live and dead HUVECs were tested using the device. HUVECs were chosen because they are widely used in studies of vascular pathology, angiogenesis and tissue engineering (Onat et al. 2011; Bishop et al. 1999; Mohr et al. 2017; Medina-Leyte et al. 2020).

Before the test, we conducted an AC frequency sweep ranging from 60 kHz to 115 kHz on a live HUVEC. At 75 kHz, the Δθ and ΔV_AC_/V_AC_ were at their maximum. Hence, we used 75 kHz as the AC excitation frequency. Details of this sweep test can be found in Supplemental Information.

**Fig. 2:**
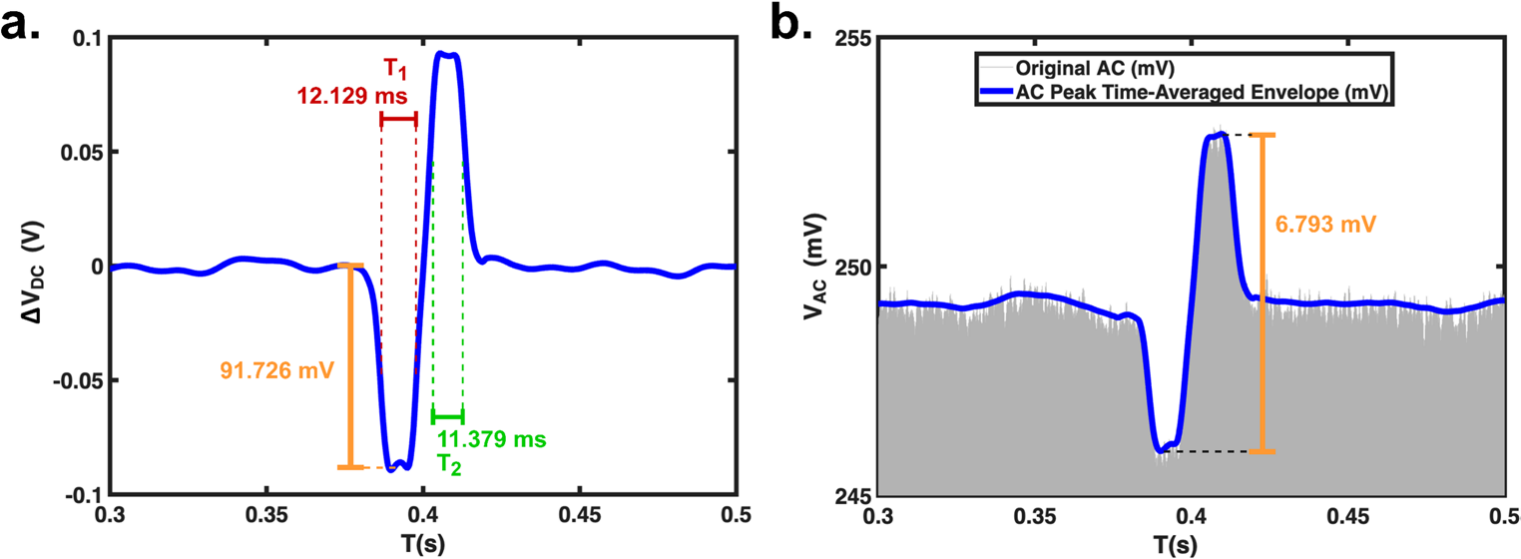
Typical DC and AC components of output voltage induced by one HUVEC passing through the sensing channels. **a**). ΔV_DC_ component, **b)**.V_AC_ component

After selecting the AC frequency, each batch of HUVECs was divided into two groups. One group was evaluated as live cells, while the other was treated with ethanol for 5 min to induce cell death; all ethanol-treated cells were confirmed to be nonviable using the Trypan Blue exclusion assay. In this study, dead cells were generated solely by ethanol treatment. The viability of each batch was assessed using the Trypan Blue exclusion assay, and the results are summarized in Table 1. Upon confirmation of viability, the cell sample was then tested. AC and DC components of the output voltage were measured as cells passed the sensing channel. A Fast Fourier Transform (FFT) algorithm was used to separate the AC component from the DC component. For the DC component, a moving average for the peak voltages was utilized. Figure 2a and b show the AC and DC components of one typical voltage pulse when a live HUVEC passes through. The negative pulse and positive pulse were generated in the 1 st and 2nd sensing channels respectively. From the DC component analysis, 1/T_2_−1/T_1_ and ΔR/R were obtained; similarly, the ΔV_AC_/V_AC_, and Δθ were obtained from the AC component. Note that T_1_ and T_2_ was defined as half-magnitude pulse width (see illustration in Fig. 1d), as suggested by a prior study (Ni et al. 2020).

Given the applied pressure of 1 kPa and the channel dimensions, the flow rate was estimated to be 2.71$\:\times\:$10^−10^ m^3^/s. For all tests, the concentration of HUVECs and hMSCs were approximately 12,500cells/mL. Per DC resistive pulse measurement (a typical pulse is shown in Fig. 2a), a cell took about 20 – 40 ms to pass through the two sensing channels. Within the 20 – 40 ms transit time range, we estimated approximately only 0.069 – 0.136 cell had a chance to be present in the sensing channel. Hence the chance that two or more cells were present in the sensing channel simultaneously would be rare.

**Fig. 3:**
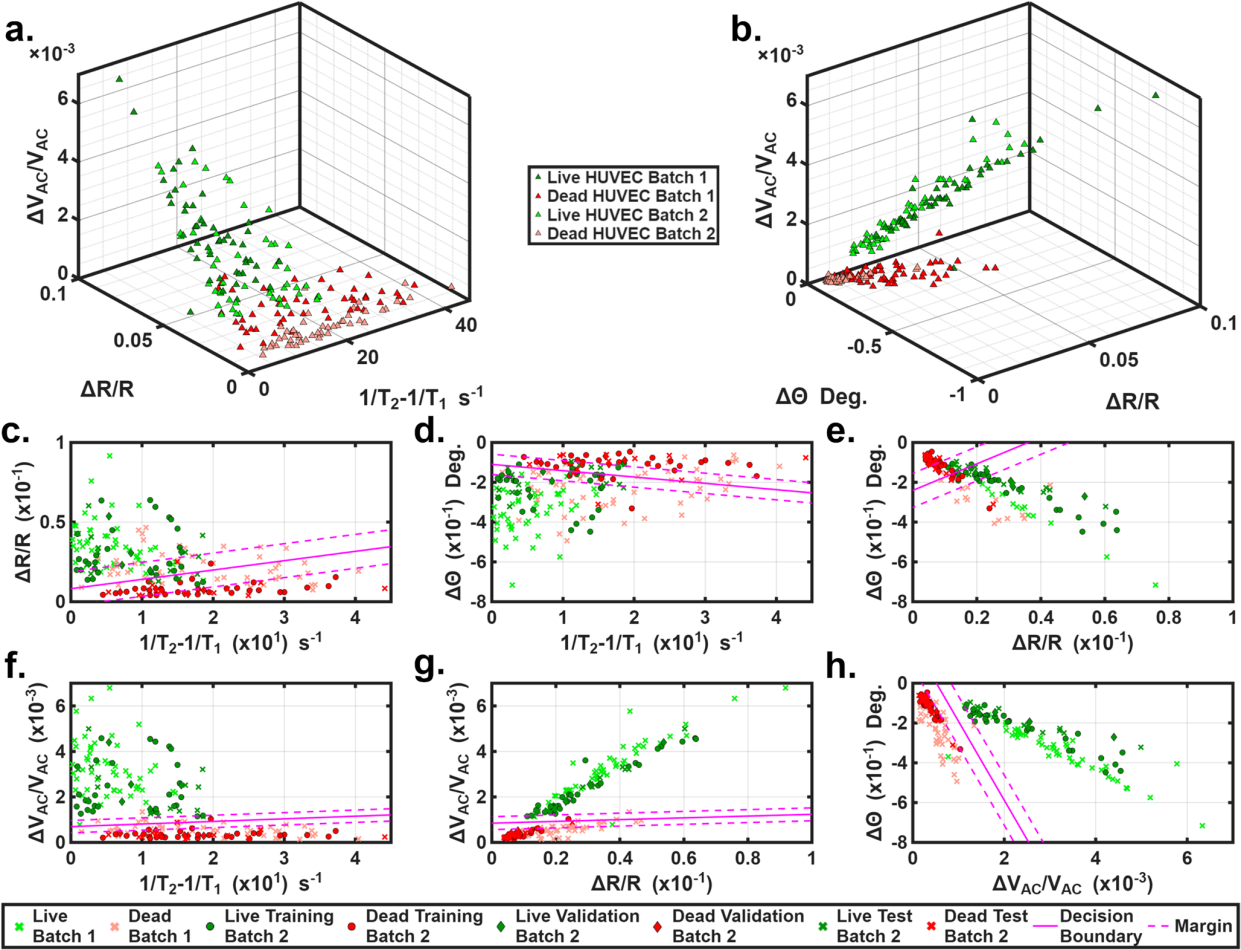
**a**). 3-D scatter plot of the ΔV_AC_/V_AC_, ΔR/R, and 1/T_2_−1/T_1_ generated by dead and live HUVECs. **b**). 3-D scatterplot of dead and live HUVECs via the ΔV_AC_/V_AC_, ΔR/R, and Δθ generated by dead and live HUVECs. **c-h**). 2-D classifications of live vs. dead HUVECs via different binary combinations of the 1/T_2_−1/T_1_, ΔR/R, Δθ, and ΔV_AC_/V_AC_. Decision boundary was generated using Batch 2 dataset

Live HUVECs and dead HUVECs were tested separately, with two independent batches of 50 cells tested for each group. The mean and standard deviation of the 4 parameters, 1/T_2_−1/T_1_, ΔR/R, Δθ, and ΔV_AC_/V_AC_ are summarized in Table 1. Figures 3c-h show 2-D scattered plots of the various binary combinations of the four parameters. Several trends were observed. Live HUVECs showed higher ΔR/R values than dead cells, indicating that live cells were larger in size. This size difference was also confirmed by microscopic observation. This finding is corroborated by Yuan et al. (2025) who observed shrinkage occurring in pyroptotic HUVECs. Further, live HUVECs showed higher Δθ values than dead HUVECs. As Δθ is indicative of cell membrane capacitance, our measurements follow the trend observed by Favakeh et al. (2025), who found that live yeast cells typically have higher membrane capacitance than dead cells. Live HUVECs induced higher ΔV_AC_/V_AC_ responses while dead HUVECs induced lower ΔV_AC_/V_AC_ values. Gawad et al. (2001) observed similar trend in ΔV_AC_/V_AC_ with live and ghost erythrocytes cells under AC excitation. Similarly, Liu et al. (2018) show that lower cell impedance for hypoxic red blood cells and a higher impedance for normoxic red blood cells across three AC excitation frequencies. Dead cells tended to have larger (1/T_2_−1/T_1_) values, indicating their zeta potential was larger than live cells. This finding was echoed by Bondar et al. (2012) who found a negative shift in zeta potential in apoptotic HeLa cells. Table 1 showed that live and dead cells had significantly different average values in each of the four parameters when assessed by a student’s t-test. However, due to the requirements of live single cell classification and natural biological variation (i.e. changes in cellular size and properties throughout cell cycle), it is difficult to use a single parameter to categorize the dead and live cells due to overlapping in parameter measurements. Once we grouped the 4 parameters into sets of two, live and dead cells formed distinctively different clusters. For example, Fig. 3a shows a 3-D scatter plot of live and dead cells in terms of the ΔV_AC_/V_AC_, ΔR/R and 1/T_2_−1/T_1_; Fig. 3b shows a 3-D scatter plot of the ΔV_AC_/V_AC_, ΔR/R and Δθ. Distinctive clusters between the dead and live samples are formed especially in Fig. 3b. Overlap was found between the live and dead clusters in Fig. 3a.

**Table 1:** Summary of multiparametric measurements, dataset partitioning and comparison of classification result to Trypan blue exclusion assay (HUVECs)

1/T_2_−1/T_1_ (in 1/s)	Dead (Batch 1)	Live (Batch 1)	Dead (Batch 2)	Live (Batch 2)
	18.8271 ± 10.1289	5.4974 ± 4.2231	18.3632 ± 9.6172	8.9041 ± 6.0088
ΔR/R	0.0217 ± 0.0098%	0.0358 ± 0.0147%	0.0080 ± 0.0044%	0.0295 ± 0.0148%
Δθ (in degrees)	−0.2294 ± 0.1037	−0.3480 ± 0.1339	−0.1126 ± 0.0549	−0.2110 ± 0.0878
ΔV_AC_/V_AC_	0.0006 ± 0.0003%	0.0032 ± 0.0012%	0.0003 ± 0.0002%	0.0023 ± 0.0011%
# cells for training	30	30	30	30
# cells for validation	5	5	5	5
# cells for testing	15	15	15	15
Classify. result (Single batch)	93.3% dead	93.3% live	100% dead	100% live
Classify. result (Cross batch)	100% dead	100% live	98% dead	98% live
Trypan Blue Assay Viability	100 ± 0% dead	99 ± 0.82% live	100 ± 0% dead	97.5 ± 0.5% live

To further classify live and dead cells, a linear support vector machine (SVM) method was used to analyze the differences in the 4 key parameters for each batch (one group of dead HUVECs sample and one group of live HUVECs sample). MATLAB was used for SVM analysis. Each batch contained 50 cells; 30 were used for training, 5 for validation (for adjusting box constraint), and 15 for testing. Details of the sample partition strategy used for SVM analysis are listed in Table 1. Figures 3c-h show the binary classifications of the Batch 2 dataset from the SVM analysis with different binary combinations of 1/T_2_−1/T_1_, ΔR/R, Δθ, and ΔV_AC_/V_AC_. The soft margin SVM generated hyperplane that acted as the decision boundary (solid pink line) in Figs. 3c-h and the upper and lower margin lines (pink dashed lines). Using the decision boundary, two distinct clusters (i.e. the live/dead cell clusters) can be classified.

From Figs. 3c-e, it can be seen that while the device was able to discern a difference in the 1/T_2_−1/T_1_, ΔR/R and Δθ values between the live and dead HUVECs, there were large overlaps between the two clusters; the margin width was quite wide with respect to the spread of the measurement data. Hence, these three binary combinations and their decision boundaries were not used for classification. Figures 3f-h shows SVM analysis of the remaining binary combinations. It can be observed from Figs. 3f-h that the live and dead HUVECs formed distinctive clusters with small overlaps. The decision boundary is well-defined, and the margin width is narrow relative to the data spread, indicating that these represent optimal combinations for distinguishing live and dead HUVECs. Figure 3h shows the best classification result among the three plots. For the 15 test cells within Batch 2, 100% of live cells and 100% of dead cells were correctly classified by the decision boundary. All of the identified live/dead cells fell outside the margin area, indicating high-confidence predictions. Hence this binary combination (Δθ and ΔV_AC_/V_AC_) and their decision boundary were chosen as the classification criteria. To verify the classification across batches, we plotted the Batch 1 dataset in Figs. 3 as light red and light green cross marks. Note that the Batch 1 dataset did not participate in training here. The classification results were 98% for both live and dead cells in Batch 1even when the decision boundary from Batch 2 was used. These classifications results matched well with the Trypan Blue assay measurement as shown in Table 1.

Next, the Batch 1 dataset was used to train the SVM model. The results were given in the Supplemental Information (Fig. S3a). The classification results within the Batch 1 dataset were 93.3% and 93.3% for live and dead cells respectively. We also conducted a cross check by using decision boundary from Batch 1 to classify all cells in Batch 2 of which 100% classification ratios were found for both live and dead cells. In Fig. S3b the decision boundaries and margins from both the Batch 2 and Batch 1 datasets were plotted together. While there were small shifts in the decision boundaries, the classification results remained nearly identical. The above cross-batch check results for HUVECs indicate reasonable stability and reproducibility of the SVM classification across independent experiments. All obtained classification results were in good agreement with those obtained from Trypan Blue assay (see Table 1).

The plots suggest that the ΔV_AC_/V_AC_ (indicative of cell impedance) is one effective parameter in classifying live and dead HUVECs. It is worth noting here that this observation is cell-type specific and different types of cells may behave differently. The dominating factor may vary or may not exist for different cell types. One advantage of this device is that it provides multi-parametric measurements. Using multi-parametric SVM analysis, it generates decision boundaries that improve classification accuracy.

### Differentiating Live/Dead hMSCs

**Table 2:** Summary of multiparametric measurements, dataset partitioning and comparison of classification result to Trypan blue exclusion assay (for hMSCs)

1/T_2_−1/T_1_ (in 1/s)	Dead (Batch 1)	Live (Batch 1)	Dead (Batch 2)	Live (Batch 2)
	9.4288 ± 4.4254	5.9680 ± 3.4503	11.7093 ± 6.8365	4.6332 ± 2.7575
ΔR/R	0.0184 ± 0.0087%	0.1011 ± 0.0372%	0.0132 ± 0.0048%	0.1180 ± 0.0430%
Δθ (in degrees)	−0.0639 ± 0.0296	−0.3136 ± 0.1319	−0.0702 ± 0.0276	−0.4481 ± 0.1505
ΔV_AC_/V_AC_	0.0013 ± 0.0006%	0.0070 ± 0.0026%	0.0011 ± 0.0004%	0.0106 ± 0.0040%
# cells for training	30	30	30	30
# cells for validation	5	5	5	5
# cells for testing	15	15	15	15
Classify. Result (Single batch)	93.3% dead	100% live	100% dead	100% live
Classify. result (Cross batch)	100% dead	100% live	98% dead	98% live
Trypan Blue Assay Viability	100 ± 0% dead	98.00 ± 0.82% live	100 ± 0% dead	99.25 ± 0.5% live

**Fig. 4:**
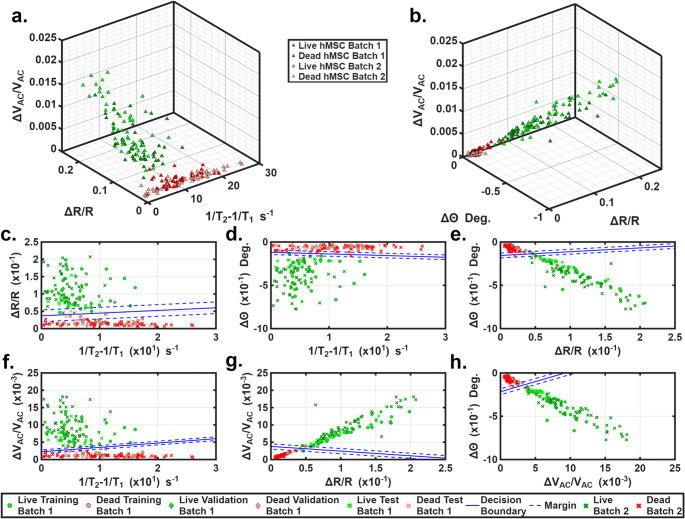
**a**). 3-D scatter plot of ΔV_AC_/V_AC_, ΔR/R, and 1/T_2_−1/T_1_ generated by live and dead hMSCs. **b**). 3-D scatterplot of ΔV_AC_/V_AC_, ΔR/R, and Δθ generated by live and dead hMSCs. **c-h**). 2-D classifications of live vs. dead hMSCs via different binary combinations of 1/T_2_−1/T_1_, ΔR/R, Δθ and ΔV_AC_/V_AC_. Decision boundaries were generated using Batch 1 dataset

We further validated the capability of our device to measure cell viability using a different cell type. hMSCs were chosen because of their widespread use in regenerative medicine and tissue engineering (Aldahmash et al. 2012). Dead hMSCs were induced solely by ethanol exposure as described previously in Section 2.3. Two batches of live and dead hMSCs were prepared and tested separately. Details are given in Table 2. The viability of each batch was assessed by Trypan Blue exclusion assay; results are presented in Table 3.2. The testing setup and data collection were the same as those used for HUVECs. Figure 4a and b show 3-D scatter plots of live and dead cells. Figure 4a uses the ΔV_AC_/V_AC_, ΔR/R, and 1/T_2_−1/T_1_ as 3 axes, while Fig. 4d uses the ΔV_AC_/V_AC_, ΔR/R, and Δθ. Distinct clusters of live and dead hMSCs can be observed in both plots with minimal overlaps. 2-D scatter plots of binary combinations of the 4 parameters are shown in Figs. 4c-h. Measurements showed distinct differences in ΔV_AC_/V_AC_, ΔR/R, and Δθ between live and dead hMSCs; however, small overlaps still existed. Although the average (1/T_2_−1/T_1_) value differed substantially between live and dead hMSCs still showed a large difference, the overlap between the two groups was large.

SVM analysis was also applied here to improve the classification. The dataset partitioning strategy is provided in Table 2. Figures 4c-h show the binary classifications of live and dead hMSCs using soft margin SVM. Decision boundaries were generated with the Batch 1 dataset. Among the 6 binary combinations, Figs. 4f-h generated good classification performance. All three plots display a clear decision boundary with narrow margins, allowing for reliable identification of live and dead cells. Figure 4g shows the best performance where the decision boundary correctly classified 100% of live cells and 93.3% of dead cells, with all identified live cells and dead cells considered highly confident. Figure 4f and h show similar result: 100%/93.3% (live/dead)for both sub-plots with 100%/92.9% (live/dead) of cells falling into the highly confident category. We chose Fig. 4g and its decision boundary for classifications. To check the classification accuracy across batches, we plotted the Batch 2 dataset in Fig. 4 as dark red and dark green cross marks. The classification remained at 100% for both dead and live cells in Batch 2 even when the decision boundary from Batch 1 was used.

Next, the Batch 2 dataset was used to train the SVM model. The results are presented in the Supplemental Information (Fig. S4a). The classification results within the Batch 2 dataset were 100% for live and dead cells. We also conducted a cross check by using the decision boundary from Batch 2 dataset to classify all cells in Batch 1, and 98% classification ratios were found for both live and dead cells. In Fig. S4b the decision boundaries and margins from Batch 1 and Batch 2 datasets were plotted together. While there were small shifts in the decision boundaries, the classification results remained nearly identical. The above results indicate reasonable stability and reproducibility of the SVM classification across independent experiments. All obtained classification results agreed well with those obtained from the Trypan Blue assay (see comparison in Table 2).

### Testing of mixed Live/Dead hMSCs

**Fig. 5:**
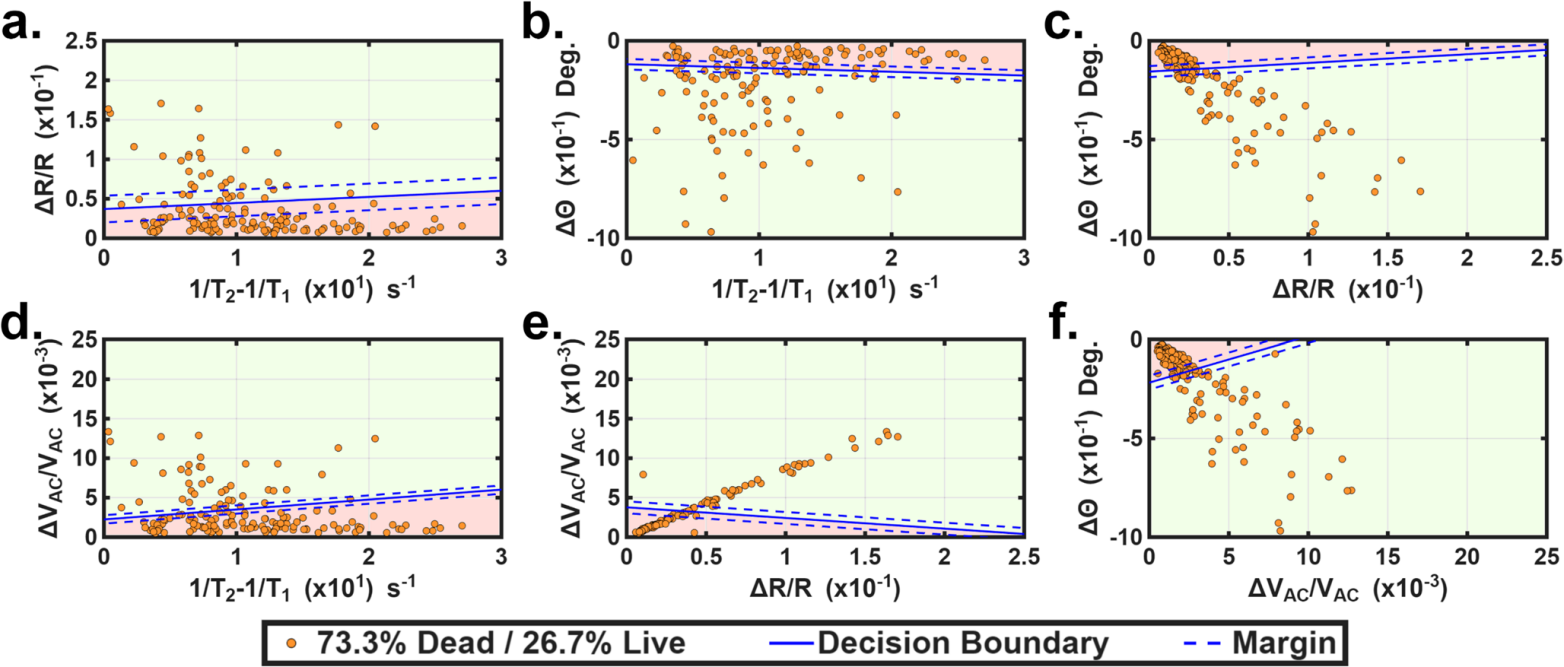
**a**-**f**). 2-D plots of binary combinations of 1/T_2_−1/T_1_, ΔR/R, Δθ and ΔV_AC_/V_AC_ measured from one 73.3%/26.7% dead/live mixed hMSCs sample. Dead/live cells were classified by the decision boundaries, which were taken from Fig. 4. Regions with background colors in red and green represent dead and live cells

Next, we conducted an experiment to demonstrate that the device can distinguish between live and dead cells in a mixed population. Live and dead cells were prepared using the same culture methods as in Section 2.3. A mixed sample was created by mixing the live/dead cells. Note that all cells from the mixed sample used in this test were prepared separately from independent culture batches and were not used in obtaining the decision boundary. In this test, dead cells were induced solely by the ethanol treatment. The mixing ratio, confirmed by Trypan Blue exclusion, was 26.7 ± 3.4% live cells and 73.3 ± 3.4% dead cells. Note that inherent variability in cell viability and mixing can lead to deviations from the intended ratio. This sample was then tested using the microfluidic device. Figure 5 shows the 2-D plots of the cells’ 4 key properties (1/T_2_−1/T_1_, ΔR/R, Δθ, and ΔV_AC_/V_AC_) obtained from the device measurements. 100 data points from cells of the mixed sample were included. The decision boundary and the margin lines were taken from Fig. 4 (i.e. obtained from the Batch 1 dataset in Fig. 4), which served as the training set by classifying a dead cell region (in red) and a live cell region (in green). In each subfigure, data points in the green region were classified as live cells, while those in the red region were classified as dead cells. We used the binary combination of ΔV_AC_/V_AC_ and 1/T_2_−1/T_1_ (shown in Fig. 5e) and its decision boundary copied from Fig. 4g as the classifier. Based on these counts, Fig. 5e shows live/dead ratios of 33%/67%. We also used the decision boundary from the Batch 2 dataset to classify the Batch 1 dataset. The results were plotted in Fig. S5 in the Supplemental Information. A similar classification ratio of 34%/66% was identified as live/dead cells. Figure 5d gives nearly identical classification results. Given that Trypan Blue exclusion typically reports accuracies of 90–95% under standard conditions and a coefficient of variation of 4%–10% depending on operator technique and sample handling (Cadena-Herrera et al., 2015; Piccinini et al. 2017; Vembadi et al. 2019), our measurement agreed reasonably well with this established method. The capability of differentiating live and dead cells from a mixture is critical for many applications including evaluating the efficacy of cancer therapies, monitoring the progression of cell-based treatments, assessing cytotoxicity of new drugs or biomaterials, optimizing tissue engineering and regenerative medicine strategies, and ensuring the quality of cell therapy products (Mirzayans et al. 2018; Giri & Galipeau 2020; Cai et al. 2024).

### Differentiating HUVECs with different treatments

**Table 3:** Summary of multiparametric measurements, dataset partitioning and comparison of classification result to Trypan blue exclusion assay and cellular morphological analysis for ethanol-treated and STS-treated HUVECs

1/T_2_−1/T_1_ (in 1/s)	Ethanol- (Batch 1)	STS- (Batch 1)	Ethanol- (Batch 2)	STS- (Batch 2)
	18.8271 ± 10.1289	7.8754 ± 3.9646	18.3632 ± 9.6172	8.1372 ± 5.6530
ΔR/R	0.0217 ± 0.0098%	0.0403 ± 0.0258%	0.0080 ± 0.0044%	0.0377 ± 0.0191%
Δθ (in degrees)	−0.2294 ± 0.1037	−0.2449 ± 0.1611	−0.1126 ± 0.0549	−0.2562 ± 0.1245
ΔV_AC_/V_AC_	0.0006 ± 0.0003%	0.0022 ± 0.0014%	0.0003 ± 0.0002%	0.0022 ± 0.0011%
# cells for training	30	30	30	30
# cells for validation	5	5	5	5
# cells for testing	15	15	15	15
Classify. result (Single batch)	100% dead	100% dead	100% dead	93.3% dead
Classify. result (Cross batch)	98% dead	96% dead	98% dead	98% dead
Benchmark viability*	100 ± 0% dead	98.36 ± 0.68% dead	100 ± 0% dead	98.9 ± 0.61% dead

*Viability result for ethanol-treated HUVEC were obtained from a Trypan Blue assay, while result for STS-treated HUVEC were obtained from cellular morphological analysis

**Fig. 6:**
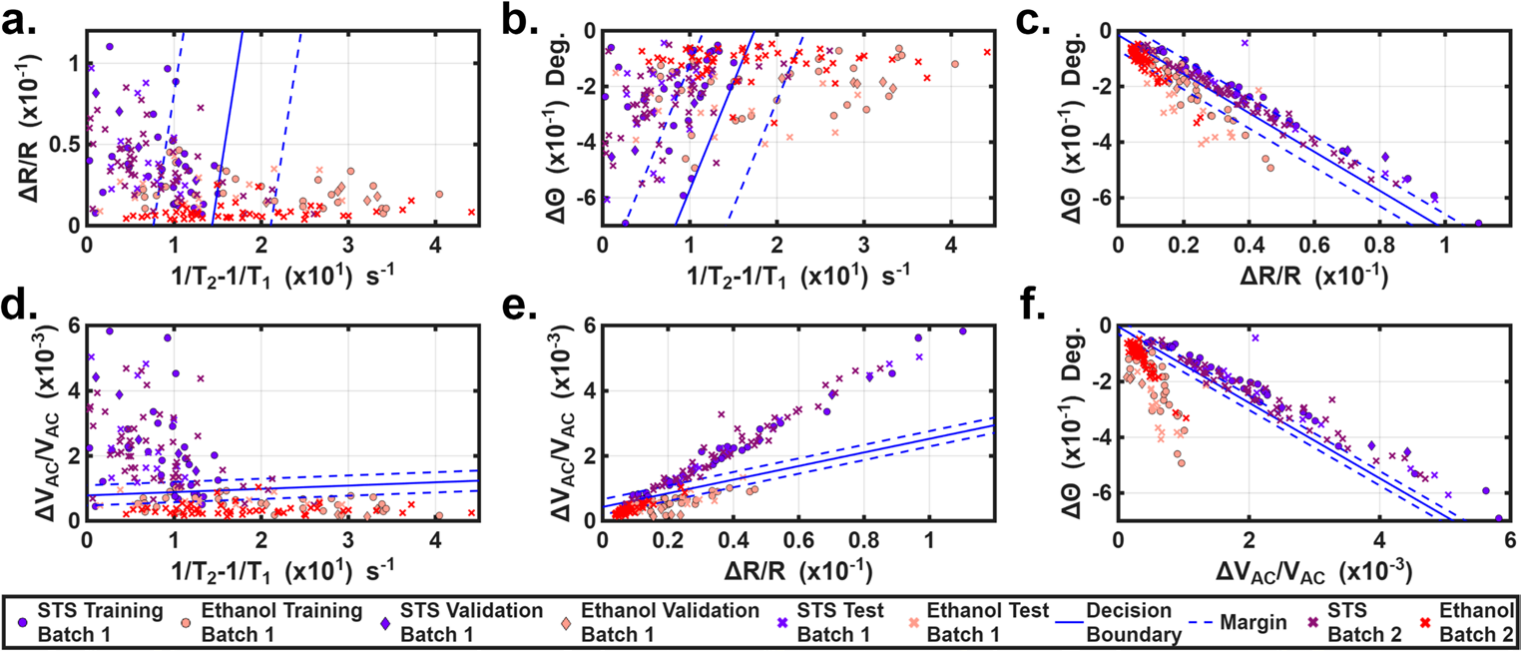
**a**-**f**). 2-D classifications of ethanol treated vs. STS treated HUVECs via different binary combinations of 1/T_2_−1/T_1_, ΔR/R, Δθ, and ΔV_AC_/V_AC_. The Batch 1 dataset was used to generate the decision boundaries

Beyond classifying live and dead cells, we next evaluated the device’s ability to differentiate between necrotic and apoptotic cell death. HUVECs from the same culture were divided into two groups: one treated with ethanol to induce necrosis, and the other treated with STS to induce apoptosis, as described in Section 2.3. Two independent batches of ethanol- and STS-treated cells were prepared. Following treatment, cell viability was assessed using the Trypan Blue exclusion assay for ethanol-treated cells and cellular morphological analysis for STS-treated cells. Ethanol- and STS-treated samples were tested separately. The datasets generated from ethanol-treated HUVECs and the STS-treated HUVECs were used to train, validate and test the SVM model separately for classifying the two treatment specific groups. Figures 6a-f show the 2-D classifications of both samples via different binary combinations of 1/T_2_−1/T_1_, ΔR/R, Δθ and ΔV_AC_/V_AC_. Soft SVM was used to generate the decision boundaries and margins using Batch 1 dataset for training, validation and testing. From Fig. 6, ethanol treated (in red) and STS treated cells (in purple) were classified. Among the four parameters, although they have large differences in the average between the two groups, there were large standard deviations and across-batch differences due to biological variances. It is difficult to use a sole dominating factor for differentiation because of the large overlaps that exist between the two cell groups. However, SVM analysis of certain binary combinations (e.g. Figure 6e, and 6f) seemed to have distinctive decision boundaries and narrow margins for the spread of the data. From Figs. 6e, f, e and f and 100%/93.3% (ethanol/STS) and 100%/100% (ethanol/STS) of cells were correctly classified for ethanol- and STS- treated cells within the Batch 1 dataset. Notably, the Δθ–ΔV_AC_/V_AC_ parameter combination shown in Fig. 6f achieved maximum accuracy, with 100% of ethanol-treated cells and 100% of STS-treated cells being correctly identified. Hence, we choose the decision boundary from Fig. 6f as the classifier. To check the classification accuracy across batches, we plotted the Batch 2 dataset in Figs. 6 and 96% of ethanol-treated cells and 98% of STS-treated cells were correctly identified, which matched well with the viability from benchmark analysis shown in Table 3.

Additionally, the Batch 2 dataset was used to train the SVM model. The results are given in Supplemental Information (Fig. S6a). Classification results within the Batch 2 dataset were 100% and 93.3% for ethanol-/STS- induced HUVECs. We also conducted a cross check by using the decision boundary from Batch 2 dataset to classify all cells from Batch 1, 98%/98% classification ratios were found for ethanol/STS induced cells. In Fig. S6b the decision boundaries and margins from the Batch 1 and Batch 2 datasets were plotted together. While there were small shifts in the decision boundaries, the classification results remained nearly identical. This cross-batch check, along with the cross-batch tests conducted for the HUVECs and hMSCs tests, confirmed that the device and the associated SVM method can classify HUVECs from different treatments with excellent accuracy; the stability and reproducibility of the classification performance across the batches is decent. All obtained classification ratios were in good agreement with those obtained from benchmark analysis (see Table 3).

### Discussion

We demonstrated a microfluidic device coupled with a soft SVM method to distinguish live and dead cells. For the two cell types tested, HUVECs and hMSCs, maximum identification accuracy reached up to 100% for both live and dead cells. In mixed hMSC samples, the measured viability agreed reasonably well with the Trypan Blue exclusion method. This approach provides high identification accuracy without requiring complex programming or being highly dependent on operator skill. Because it is based on the physical characteristics of cells, the method is non-destructive and minimally affects cell function. Consequently, it enables repeated viability measurements on the same sample and allows the measured cells to be used for downstream applications, which are valuable for longitudinal studies, drug screening, tissue engineering, and other applications where preserving cell integrity is critical. Note that we conducted SVM analysis on independent datasets from different sample batches. These datasets were collected across different runs, days and devices. Although a slight shift in the decision boundary was observed, the classification accuracy remained nearly identical, suggesting good stability and reproducibility of the SVM classification across independent experiments.

Compared to microfluidic multispectral approaches using high frequencies (i.e. 500 kHz-50 MHz) (Sui et al. 2020; Gawad et al. 2001; McGrath et al. 2020; Heidmann & Di Berardino 2017; Spencer et al. 2020; Eades et al. 2023), this method needs only DC and low frequency AC excitations, thus significantly reducing the hardware complexity and post data processing. Therefore, this microfluidic device can be used as a portable device for field applications including point-of-care diagnostics, rapid assessment of cell viability in biomanufacturing, on-site drug toxicity testing, environmental monitoring of microbial or mammalian cells, and quality control of cell therapy products.

While we only demonstrated differentiations of live and dead cells, the device and the associated SVM method can also be used to differentiate cell types. Many studies suggested different cell types exhibit differences in physical properties including size, shape, surface charge, membrane capacitance, impedance, etc. (Liu et al. 2020; Mogilner & Keren, 2009; Hatton et al. 2023; Ouyang et al. 2021; Kokabi et al. 2025). While the cells can be identified via antibody-based (e.g. ELISA) (Behl et al. 2006; Liu et al. 2016; Han et al. 2016), aptamer based (Li et al. 2018; Shi et al. 2011), and reporter gene based (Konopka et al. 2010; Teow et al. 2019) bio-recognitions, these methods all need tedious and lengthy surface modifications or labeling, which may not be available for field applications or harsh environments. In comparison, the device and classification method presented here are suitable for these applications with no need for surface modifications.

Within the four key parameters, although the Δθ and ΔV_AC_/V_AC_ are affected by the C_m_ and overall impedance, we did not intend to extract the cells’ specific C_m_ and R_cyto_ values. First, extracting C_m_ and R_cyto_ requires measurements at multiple frequencies; however, it is difficult to establish a model that accurately represents cellular electrical behavior over a broad frequency range. Second, precise estimation of the double-layer capacitance (C_dl_) is challenging due to the complex and dynamic electrochemical processes involved. While the use of a single low frequency limits the extraction of C_m_ and R_cyto_, our results demonstrate that combining Δθ and ΔV_AC_/V_AC_ with ΔR/R and (1/T₂ − 1/T₁) is effective for classifying different cell groups.

Prior studies showed that a specific parameter (e.g. Δθ or ΔV_AC_/V_AC_) can be used as the criterion to differentiate cells (Sui et al. 2020; Eades et al. 2023). However, the dominant factor is often cell-type specific. The dominating parameter may vary and may not exist for different cell types. Our device relies on the decision boundary generated by the multiparametric SVM analysis, which was demonstrated to have generated up to 100% classification accuracies. While the SVM generated different decision boundaries based on different combinations of parameters, the combination and its decision boundary that yields the maximum classification accuracy should be selected as the classifier. Applying feature weighing methods may further improve classification applicability and performance, which deserves further studies.

Despite the promises, several factors that may influence the classification are discussed below:

#### Differential deformation of Live/Dead cells

Cell deformation under shear stress and its impact on impedance have been previously reported (Zhou et al. 2018; Fajdiga et al. 2025). The microfluidic sensing channel used in this device is similar to those employed in shear flow deformability cytometry devices (sDCs). Studies with sDCs have shown that, regardless of cell type, cells experience measurable deformation under comparable shear conditions, with the extent of deformation varying by cell type and treatment (Xavier et al. 2016; Fajdiga et al. 2025). This cell-type dependent deformation can influence both cell size and impedance (Zhou et al. 2018). While we acknowledge that shear-induced deformation may confound impedance and size related measurements (e.g. ΔR/R, Δθ or ΔV_AC_/V_AC_), classifications based on these measurements under consistent testing conditions remain valid for assessing cell viability or distinguish treatment-specific cell populations (Ye et al. 2026; Xavier et al. 2016; Fajdiga et al. 2025). Future work could integrate an optical-based sDC with this device to quantify the effects of shear deformation and provide a richer dataset for analysis.

#### Influencing factors for the four key measurements

 While the device demonstrated the capability to accurately classify live/dead cells, it is worth mentioning that the measurements are affected by several factors: (1) flow velocity- To identify the transit time difference in terms of (1/T2−1/T1), the flow velocity cannot be too large so that the electrophoretic velocity is significant. On the other hand, if the flow velocity is too small, the throughput of the cell detection would be low. As a tradeoff, an electrophoretic velocity/flow velocity ratio of 1% to 5% seemed appropriate to generate a noticeable 1/T2−1/T1 while maintaining decent throughput. In our tests, the electrophoretic velocity was approximately 2% to 3% of the flow velocity. (2) electrolyte conductivity- It affects the double layer capacitance Cdl and the electrolyte resistance RSOL, which influences the equivalent circuit and thus Δθ and ΔVAC/VAC values. (3) temperature- Cell membrane capacitance generally increases with rising temperature due to changes in the membrane structure and ion channel behavior, which would ultimately change Δθ and ΔVAC/VAC values (Pinto et al. 2022). (4) electrode condition- Polarization of Ag/AgCl and surface contamination would affect the Cdl and introduce parasitic capacitance, which contributes to measurement errors (Seaton & Heien 2021). Careful preparation and maintenance of the electrodes are necessary. While all the above-mentioned factors could cause measurement errors, it is worth mentioning that in practical applications, the measurement and subsequent training need to be conducted under the cell’s specific culture conditions (e.g. media, temperature, etc.). The flow rate should be selected based upon the cell zeta potential so that the electrophoretic velocity is detectable.

#### Cell aggregation effect

Cell aggregates can also affect the classification result. For example, a 2- or 3-cell aggregate can produce a 2X or 3X increase in ΔR/R while causing a similar change in transit (1/T_2_−1/T_1_). Due to the heterogeneous nature of cells, such aggregates may be misidentified as a single larger cell, leading to artificially increased ΔV_AC_/V_AC_ and other measurements. This misidentification could introduce incorrect inputs into the training dataset and reduce classification accuracy. In our experiments, no cell aggregates were observed in the channels by microscopic inspection. Nevertheless, cell aggregation remains a potential issue in most live-cell cytometry applications. Non-ionic, surfactant-based pretreatment strategies (e.g. Pluronic F-68, Accumax, etc.) can be applied, as commonly done in other flow cytometry techniques when aggregation is a known concern.

#### Limits of single passage and small sample size

In this study, we tested a limited range of biological variations in terms of cell passage number, sample size and culture conditions. Only a single passage of cells was used for each cell type to ensure that the device response reflected solely the cellular response to each treatment. Standardized culture conditions were used, as they are the most common conditions for cell culture. Future work will expand to include a wider range of biological variables, such as additional cell types and passages, varied culture conditions (e.g., serum deprivation, hypoxia, or different temperatures), and larger sample sizes.

#### Cell-type dependent classification

Lastly, we note here that cell viability classification is dependent on cell type. To apply this method to new cell type, measurements should be performed under each cell’s specific culture conditions (e.g., media, temperature) and at an appropriate flow rate. SVM training, validation and should be conducted independently for each cell type under these conditions. In future work, we plan to extend the applicability of this device to additional cell types.

## Conclusions

We demonstrated a microfluidic device that can differentiate live cells from dead cells, via providing measurements of multiple physical properties. The device utilizes a low frequency AC signal with a DC bias applied across its two consecutive sensing channels. When single cells pass through the sensing channels, the DC response provides size and surface charge information in terms of measuring the resistive pulse magnitude and the transit time difference while the AC response measures the phase angle and peak voltage change, which are influenced by each individual cell’s membrane capacitance and impedance. Using the multiparametric measurement, live cells and dead cells can be differentiated. The low frequency measurement avoids complex hardware and post-processing. A soft margin support vector machine (SVM) was used to determine the decision boundary and margin for live/dead cell classification. Our microfluidic device, combined with an SVM method, reliably distinguished live from dead cells for HUVECs and hMSCs, achieving a classification accuracy up to 100%. In mixed hMSC samples, the measured viability closely matched expected ratios, demonstrating strong agreement with Trypan Blue exclusion assay results. The device also differentiated STS-treated and ethanol-treated HUVECs with accuracies up to 100%. Although cell viability classification is cell-type dependent, this method can be potentially extended to other cell types by taking separate measurements and performing SVM training under each cell type’s specific culture conditions. Despite its simple design, the device enables multiparametric single-cell measurements in continuous flow without surface modification, providing a portable platform for applications such as drug efficacy screening, evaluation of new therapies and investigation of cellular toxicity mechanisms.

## Supplementary Information

Below is the link to the electronic supplementary material.

Supplementary Material 1 (DOCX 884 KB)

## Data Availability

Data sets generated during the current study are available from the corresponding author on reasonable request.
