# Supplementary material for: In situ detection of dead cells from live cells via a DC plus low frequency AC resistive pulse sensor
Source: Biomed Microdevices. 2026 Feb 13;28(1):11. doi: 10.1007/s10544-026-00797-y (PMC12904940; doi:10.1007/s10544-026-00797-y)
Supplement: Supplementary file 1 — Supplementary Material 1 (DOCX 884 KB) [file 10544_2026_797_MOESM1_ESM.docx]

**Supplemental Information**

**1. Excitation Frequency Selection**

We aimed to select a low AC excitation frequency. This is due to the observation by Liu et al. 2018, which reported adequate differences in phase angle and impedance between live and dead cells at 156 kHz. Our target frequency range was around 100 kHz. To determine optimum excitation frequency, we measured the device’s response at various frequencies at near 100 kHz, when a cell passed through the device.


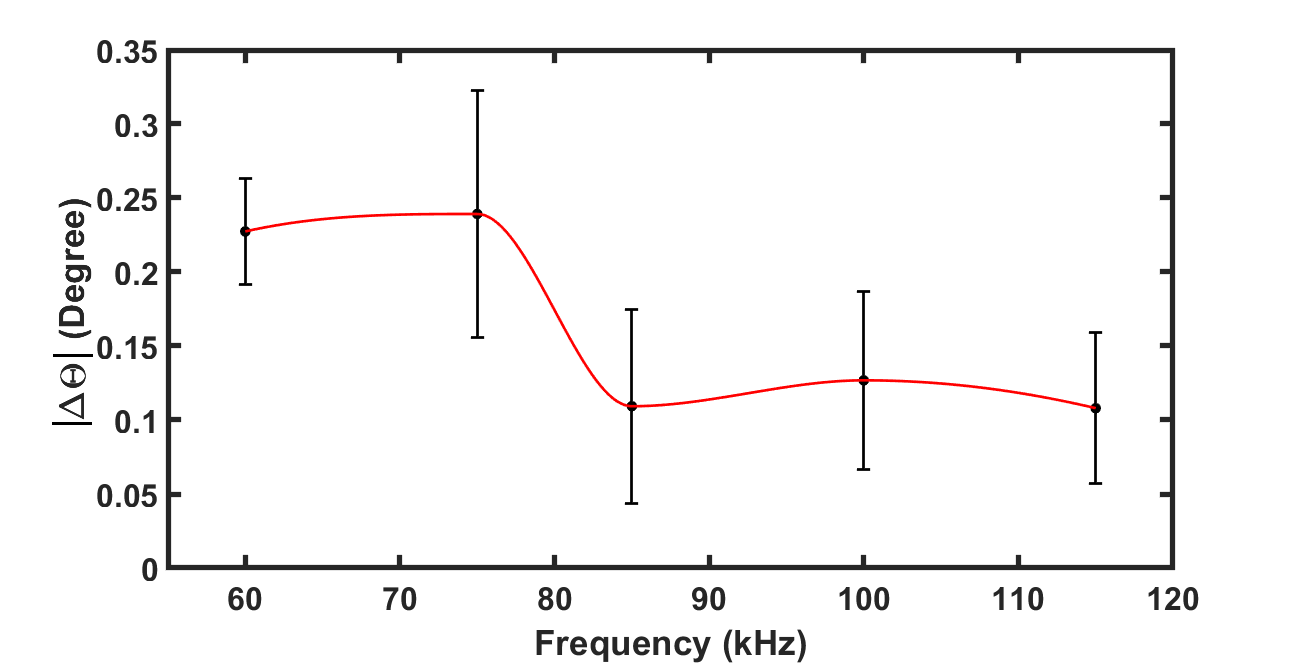


**Figure S1:** Measured Δθ induced by one HUVEC cell at frequencies from 60 kHz and 115 kHz. 5 measurements were taken at each frequency.


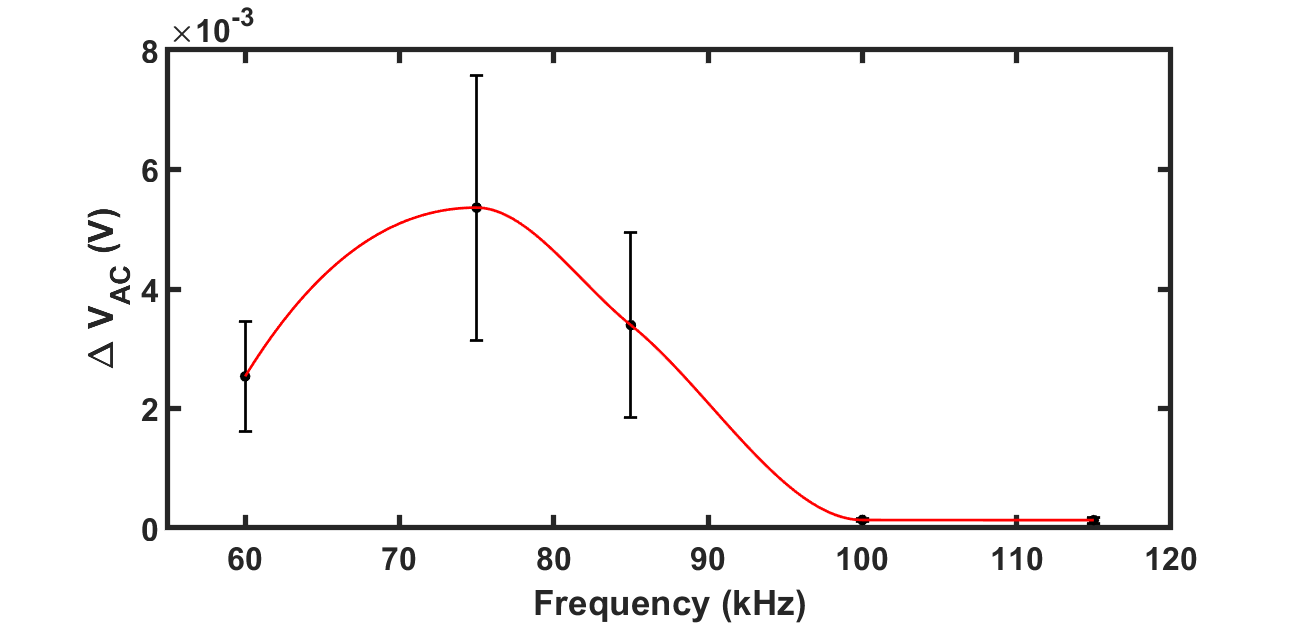


**Figure S2:** Measured ΔV_AC_ induced by one HUVEC cell at frequencies from 60kHz and 115 kHz. 5 measurements were taken at each frequency.

When a single live HUVECs passed the sensing channel, the phase angle θ change and ΔV_AC_ change at 5 distinct excitation frequencies (60 kHz, 75 kHz, 85 kHz, 100 kHz, and 115 kHz) was measured. The HUVECs was forced to pass through the sensing channels five times at each frequency; AC and DC components of the voltage response were recorded; phase angle change Δθ, and ΔV_AC_ change was analyzed. Figure S1 (Top) shows the mean ±std of phase angle θ change at each frequency. Figure S2 (Bottom) shows the mean ± std of ΔV_AC_ change. From the results, 75 kHz was selected as the AC excitation frequency as it generated the largest phase angle and magnitude change (Δθ, and ΔV_AC_) within the 60 kHz to 115 kHz range.

**2. Demonstrate stability and reproducibility of the SVM classification across independent experiments**

**2.1 Live/Dead HUVECs classification using Batch 1 dataset**


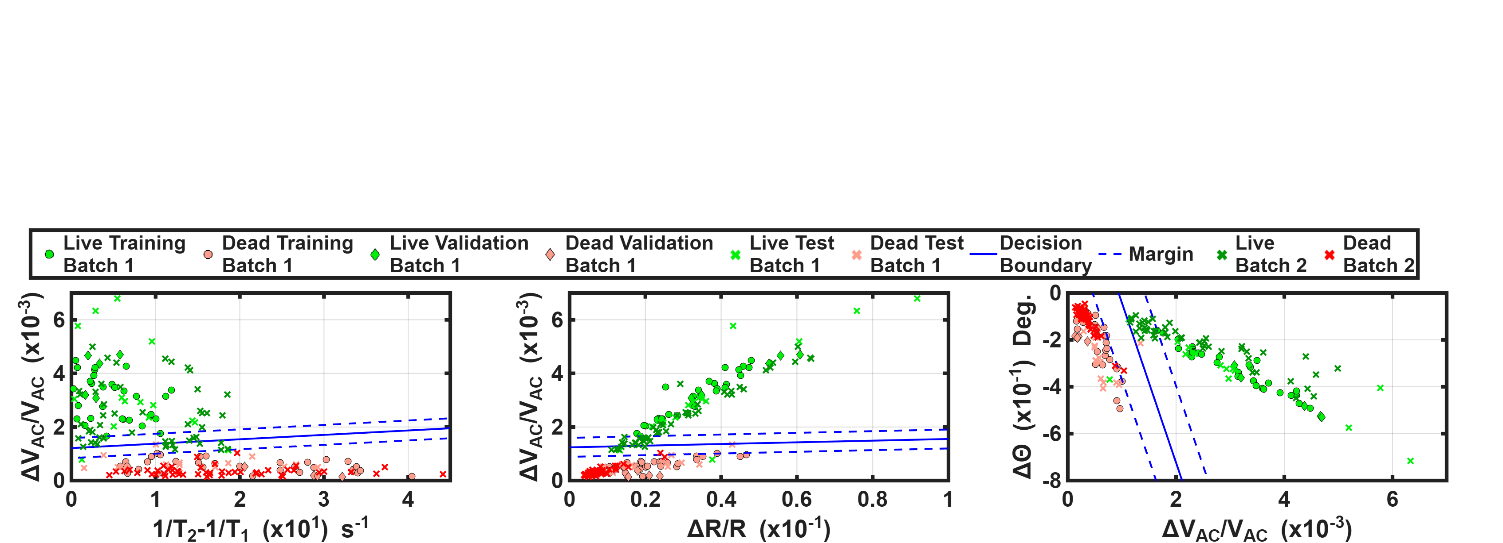


1. (ii) (iii)

Fig. S3a: 2-D classifications of live vs dead HUVECs via different binary combinations of 1/T_2_-1/T_1_, ΔR/R, Δθ, and ΔV_AC_/V_AC_ that generated good classification results. Decision boundaries were generated by Batch 1 dataset. Fig. S3a-iii) and its decision boundary was used as classifier.


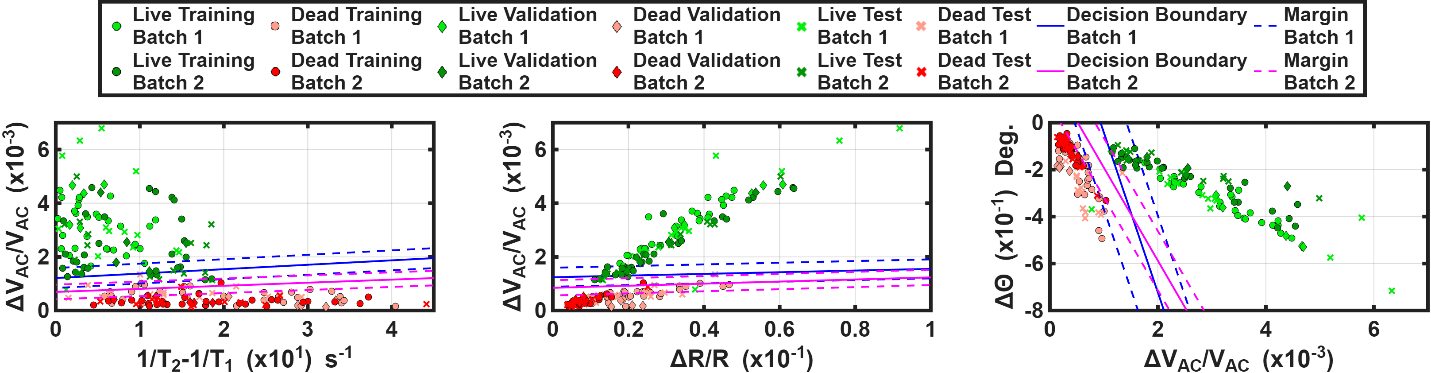


(i) (ii) (iii)

Fig. S3b: Comparison of decision boundaries from Batch 1 (blue color solid line) and Batch 2 (pink color solid line) for HUVECs.

In Fig. S3a, Batch 1 dataset was used to train the SVM model. Classification accuracies within Batch 1 dataset are 93.3% and 93.3% for dead/live cells. We also conducted a cross check by using decision boundary from Batch 1 to classify all cells in Batch 2, 100% and 100% classification ratios were found for dead/live cells. In Fig. S3b the decision boundaries and margins from Batch 2 and Batch 1 dataset were plotted together; while there were small shifts in the decision boundaries, the classifications results remained nearly identical. The above cross-batch check results for HUVECs indicate reasonable stability and reproducibility of the SVM classification across independent experiments. All obtained classification results were in good agreement with those obtained from Trypan Blue assay.

**2.2 Live/Dead hMSCs classification using Batch 2 dataset**

Similar cross-batch SVM analysis were also conducted for hMSCs.


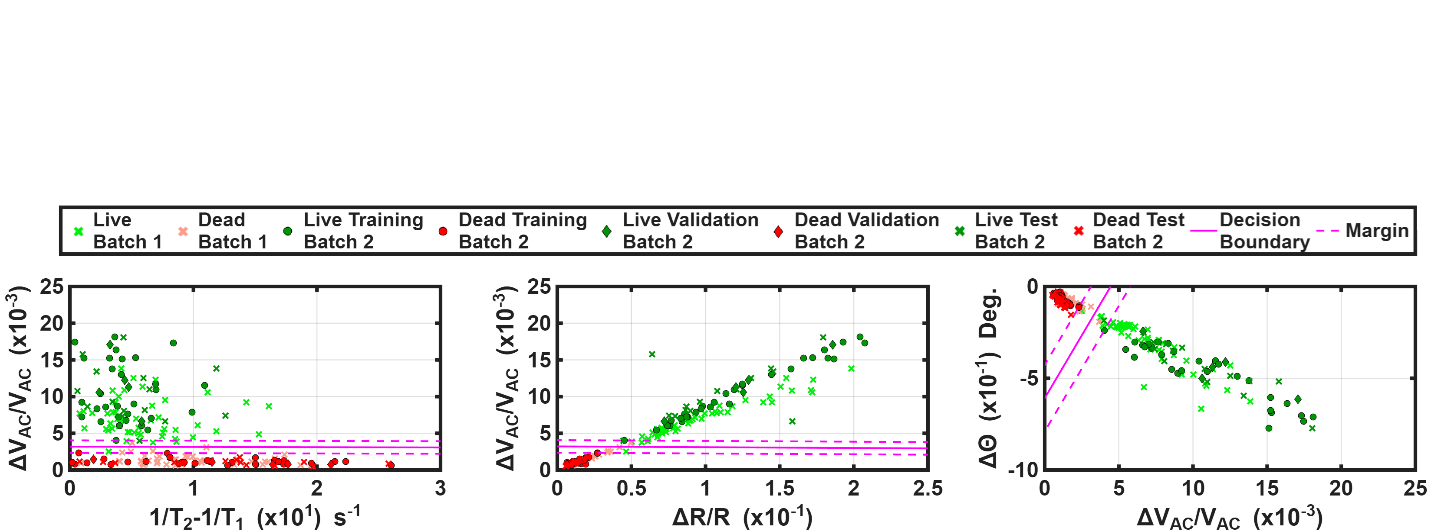


1. (ii) (iii)

Fig. S4a: 2-D classifications of live vs dead hMSCs via different binary combinations of 1/T_2_-1/T_1_, ΔR/R, Δθ, and ΔV_AC_/V_AC_ that generated good classification results. Decision boundaries were generated by dataset from Batch 2. Fig. S4a-ii) and its decision boundary was used as classifier.


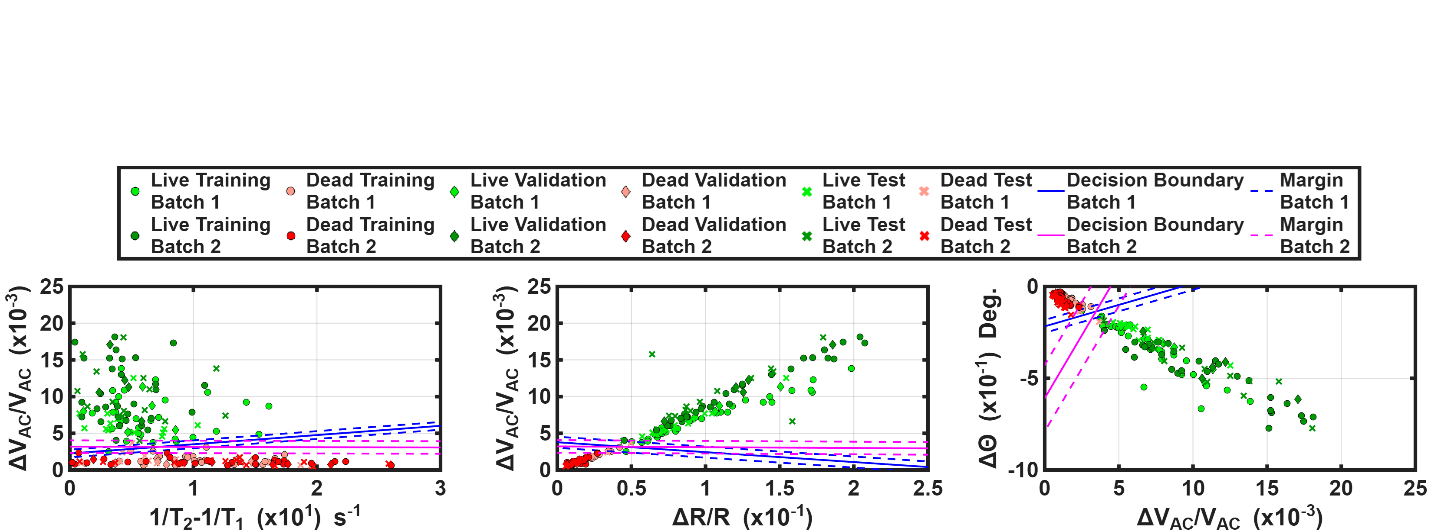


1. (ii) (iii)

Fig. S4b: Comparison of decision boundaries from Batch 1 (blue color solid line) and Batch 2 (pink color solid line) for hMSCs.

In Fig. S4a, Batch 2 dataset was used to train the SVM model. Classification accuracies within Batch 2 dataset are 100% and 100% for dead/live cells. We also conducted a cross check by using decision boundary from Batch 2 dataset to classify all cells in Batch 1, 98% and 98% classification ratios were found. In Fig. S4b the decision boundaries and margins from Batch 1 and Batch 2 datasets were plotted together; while there were small shifts in the decision boundaries, the classification results remained nearly identical. The above results indicate reasonable stability and reproducibility of the SVM classification across independent experiments. All obtained classification results agreed well with those obtained from Trypan Blue assay.

**2.3 Testing of mixed dead/live hMSCs**


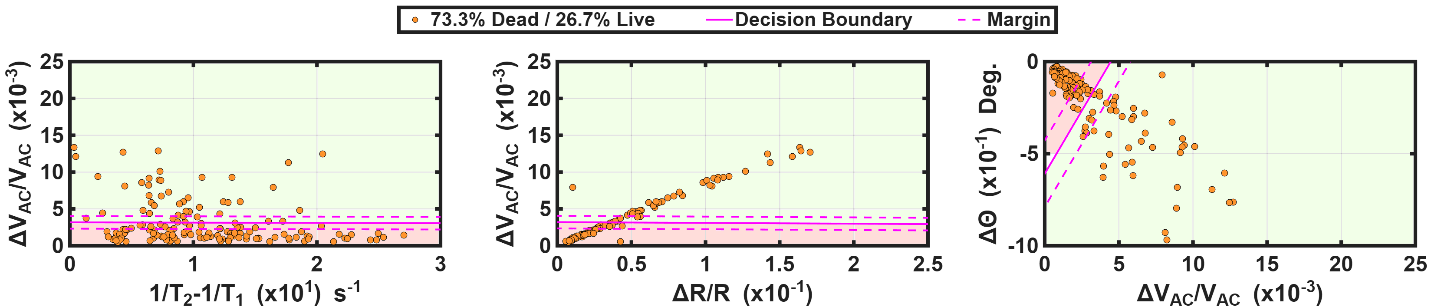


1. (ii) (iii)

Fig. S5: 2-D plots of binary combinations of 1/T_2_-1/T_1_, ΔR/R, Δθ and ΔV_AC_/V_AC_ measured from one 73.3/26.7 dead/live mixed hMSCs sample. Decision boundaries were generated by the dataset from Batch 2 of hMSCs. Decision boundary obtained from Fig. S4a-ii was used as classifier.

We used the decision boundaries from Batch 2 dataset of the hMSC test. Using the decision boundary of Fig. S4a-ii as classifier, the results shows classification ratio of 34%/66% live/dead cells. This result is similar to the classification ratio of 33%/67% live/dead ratio shown in Fig. 5e that used Fig. 4g (decision boundaries from Batch 1 dataset of the hMSC test) as the classifier for differentiation.

**2.4 Treatment-specific HUVEC cells classification**


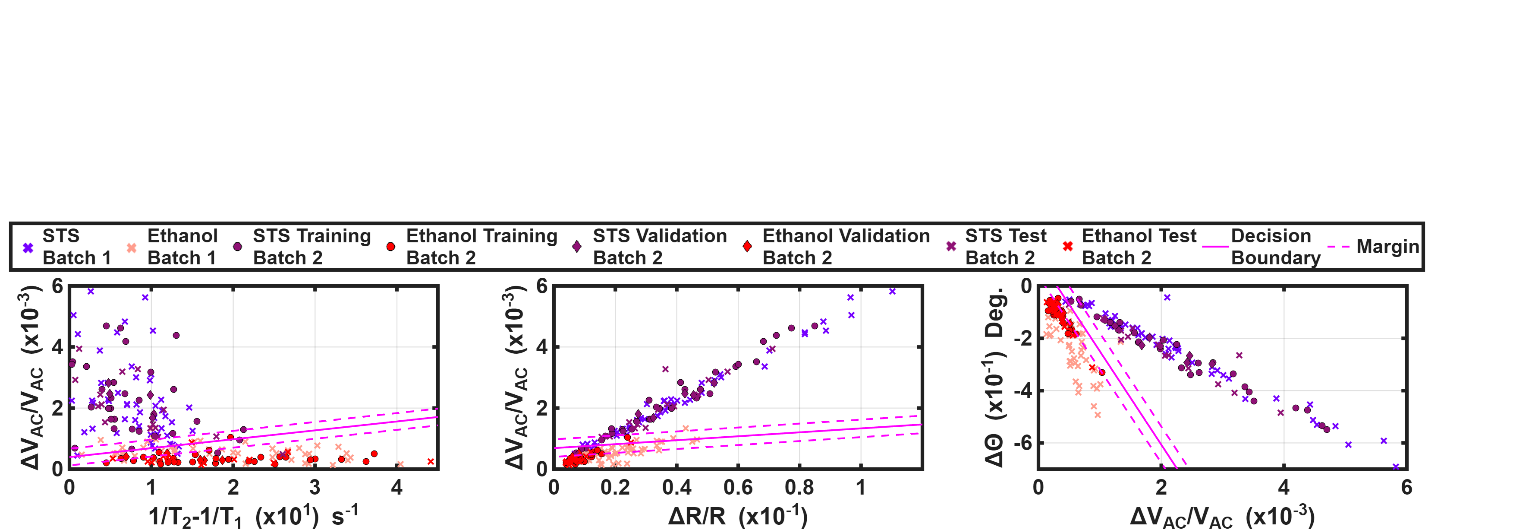


1. (ii) (iii)

Fig. S6a: 2-D classifications of ethanol- and STS- induced dead HUVEC cells via different binary combinations of 1/T_2_-1/T_1_, ΔR/R, Δθ, and ΔV_AC_/V_AC_. Decision boundaries were generated by dataset from Batch 2. Fig. S6a-iii and its decision boundary was used as classifier.


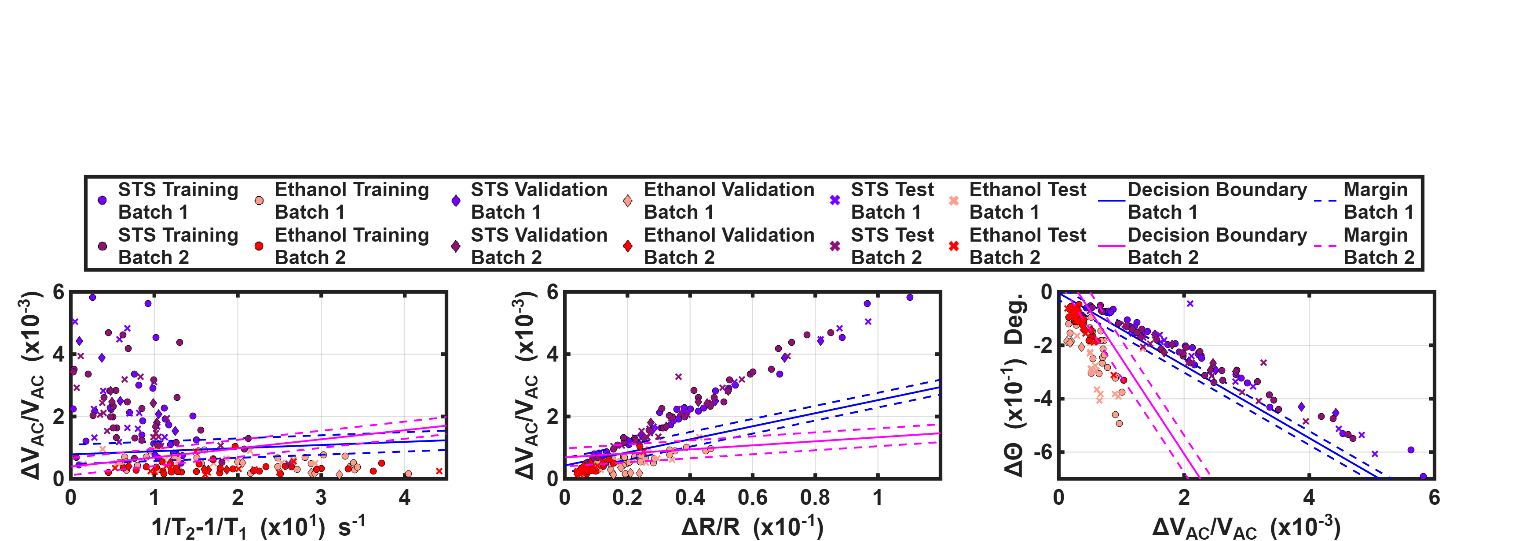


1. (ii) (iii)

Fig. S6b: Comparison of decision boundaries from Batch 1 (blue color solid line) and Batch 2 (pink color solid line) for ethanol- and STS-treated HUVEC cells.

In Fig. S6a, Batch 2 dataset was used to train the SVM model. Classification results within Batch 2 dataset were 100% and 93.3% for ethanol-/STS- induced HUVECs. We also conducted a cross check by using decision boundary from Batch 2 dataset to classify all cells in Batch 1, 98% and 98% classification ratios were found for dead/live cells. In Fig. S6b the decision boundaries and margins from Batch 1 and Batch 2 dataset were plotted together. While there were small shifts in the decision boundaries, the classification results remained nearly identical. This cross-batch check, along with the cross-batch tests conducted for HUVECs and hMSCs tests, confirmed the device and the associated SVM method can classify cells HUVECs from different treats with excellent accuracy; the stability and reproducibility of the classification performance across batches is decent. All obtained classification ratios were in good agreement with those obtained from benchmark analysis.

**3. Validate the device for cell size differentiation.**

There were two unknown factors that prevent us from directly using equation (2) to calculate the cell size: 1) the unknown F factor, and 2) the effect of the connecting channel between the electrode hole and the sensing channel. Direct measurement of cell size would need a calibration by using a series of microparticles with known sizes. By measuring the DC resistive pulse magnitude induced by each sized particles, the calibration curve can be established, from which cells’ sizes can be back calculated from their resistive pulse magnitudes. While the size measurement of resistive pulse sensor is well known, our major goal is to classify different cell groups; thus we did not conduct such a size calibration. Despite this, we add a check to validate the device’s size differentiation capability. It is well acknowledged that the resistive pulse sensor’s output (ΔR) under DC is proportional to particle’s volume, i.e. ΔR $\propto$ d^3^, where d is the particle’s diameter. In our approach, we measured the sizes of hMSCs in Batch 1 under the microscope; the diameters were 29.50 ± 6.41 μm (for live cells) vs 16.84 ± 2.61 μm (for dead cells). We also measured the ΔR/R values of the same Batch; we obtained 0.1011 ± 0.0415 (for live) and 0.0183 ± 0.0093 (for dead). The ratio of the resistance change, 0.1012/0.0184 (= 5.5) closely matched the cubic of the diameter ratio, (29.5/16.8)^3^ (=5.4). This check demonstrated that the device can accurately reflect the cells’ size changes.
